# Object‐Control Skills and Cortisol Response During Physical Education Lessons in Primary School Children

**DOI:** 10.1111/josh.70236

**Published:** 2026-09-19

**Authors:** Fioretta Silvestri, Davide Curzi, Giovanna Zimatore, Valerio Bonavolontà, Emiliane Rubat du Mérac, Silvia Migliaccio, Carlo Baldari, Laura Guidetti, Maria Chiara Gallotta

**Affiliations:** ^1^ Department of Economic, Psychological, Communication, Education and Movement Science University Niccolò Cusano Rome Italy; ^2^ Department of Theoretical and Applied Sciences eCampus University Novedrate Italy; ^3^ Department of Biotechnological and Applied Clinical Sciences University of L'aquila L'Aquila Italy; ^4^ Department of Psychology of Development and Socialization Processes Sapienza University of Rome Rome Italy; ^5^ Department of Experimental Medicine Sapienza University of Rome Rome Italy; ^6^ Department of Physiology and Pharmacology “Vittorio Erspamer” Sapienza University of Rome Rome Italy

**Keywords:** fundamental motor skills, motor coordination, physical education, psychomotor performance, stress response

## Abstract

**Background:**

Object‐control (OC) skills are fundamental movement skills acquired during primary school that may act as either stressors or engagement factors during physical education (PE). This study examined the acute impact of OC exercises on children's stress levels across grade levels.

**Methods:**

124 primary school children (Grades 3–5) participated in two PE lessons, one including OC exercises and one without. Salivary cortisol (C) was measured before and after each lesson.

**Results:**

Fourth‐grade children showed lower C concentrations than fifth‐grade students (*p* = 0.02). Following PE without OC, third‐grade C levels decreased (*p* = 0.045), fourth‐grade increased (*p* < 0.01), and fifth‐grade showed no change. Following PE with OC, C decreased only in fifth‐grade children (*p* = 0.03).

**Implications for School Health Policy and Practice:**

Because stress‐related effects of OC exercises differ by grade level, PE curricula should introduce OC tasks progressively.

**Conclusions:**

Primary school children exhibit distinct, grade‐dependent neuroendocrine responses after physical activity. High cognitive loads in complex OC tasks may buffer typical cortisol reductions in younger children, whereas mature cohorts find OC activities stress‐regulating. Evaluating motor skill development alongside hormonal adaptations can help optimize school health policies and stress management strategies.

## 1 Introduction

In Europe, primary school pupils receive a mean of 2 h of physical education (PE) per week [1]. Because of this limited time, it is crucial to identify interventions that engage children in future sport participation and support the development of their motor skills. The ability to perform fundamental movement skills, such as running, jumping, balancing, and hopping, reflects children's gross‐motor coordination, also described as their level of motor competence [2]. Italian children show a comparatively high prevalence of low or poor motor competence relative to other European countries [3]. Children's motor skills depend on several factors, including age, sex, and weight status [4], sports history [5], and environmental context [6], which together contribute to the variability in motor competence observed during childhood [5, 7]. Because the growth spurt between 8 and 10 years of age represents a critical window for gross‐motor coordination development [8], PE lessons in the final years of primary school should include coordinative exercises that target these movement skills.

Among fundamental movement skills, object‐control (OC) skills appear to be strong predictors of children's active participation in physical activity in primary school, contributing to increased daily engagement in moderate‐to‐vigorous physical activity (MVPA) [9]. Early competence in OC skills provides the foundation for the development and transfer of more advanced motor abilities, supporting increased physical activity levels and healthier lifestyles over time [10]. Because many sports activities, such as team sports, require a high level of OC ability (e.g., ball handling and ball kicking), training these more complex fundamental motor skills becomes increasingly important as children grow older, particularly during the final years of primary school [11]. However, the development of OC skills across childhood and preadolescence is not linear: analyzing 186 children aged 5–14 years, Butterfield et al. [12] described how OC abilities such as catching, throwing, and kicking progress through phases of rapid, exponential increase followed by phases of stabilization.

In the school context, OC tasks may act as a stressor for children who have not yet mastered them, potentially eliciting psychosocial stress during PE lessons. High cognitive‐control tasks, associated with poor motor coordination, have shown negative effects on both children's motor performance and self‐efficacy [13]. At this stage of life, children are still developing self‐confidence, self‐esteem, and body consciousness, and may not be aware of their own capacity to learn new, complex skills through training [14]. This mechanism may not only prevent children from benefiting from the stress‐reducing effects of physical activity [15], but could also lead them to experience a sense of self‐inefficacy.

A non‐invasive method for assessing stress levels in the youth population is measuring salivary cortisol concentrations [16, 17], an approach used in several studies involving children and adolescents to evaluate stress response following physical activity interventions. Jäger et al. [18] found that a cognitively engaging physical activity session lasting just 20 min increased salivary cortisol in primary school children. Using different types of physical activity interventions, other researchers observed that acute high‐intensity aerobic exercise did not significantly increase salivary cortisol in primary school children [19] or adolescents [20]. Other authors have reported that 30 min of physical activity, including balance, coordination, and speed exercises, can attenuate psychosocial stress‐induced cortisol increases in healthy children aged 7–11 years, suggesting physical activity may protect children's adaptability to stress [16]. According to these different findings, Wegner et al. [21] concluded that different types of physical activity intervention, such as exercises with a high aerobic component or those requiring greater coordinative effort, elicit distinct cortisol responses in children aged 9–10 years.

In the context of PE, the effects of OC tasks on children's stress response remain unclear. Cognitive load is higher when a skill is first learned and decreases as it becomes automated through motor practice [22]. Moreover, children and adolescents with poor motor coordination could experience increased psychosocial stress in social contexts such as schools [23], where peer relationships influence their psychological well‐being [24]. On the other hand, OC exercises delivered in a game‐based format increase enjoyment in children below the age of 12, compared with traditional PE [25], and ball‐skill competence is strongly positively associated with perceived motor competence and PE enjoyment [26]. Compared with locomotor tasks, OC tasks additionally require hand‐eye or foot‐eye coordination, spatial prediction, and precise timing, and demand inhibitory control, working memory, and outcome‐based adaptation: executive‐function demands not equivalently elicited by locomotor tasks alone [27]. Therefore, the aim of this study was to compare children's cortisol responses to a PE lesson without OC exercises with a PE lesson including OC exercises, and examined whether this response was associated with participants' age.

## 2 Methods

### 2.1 Participants

The study involved 124 children (59 girls and 65 boys) aged between 9 and 11 years, recruited from nine Italian primary school classes. Specifically, 39 students belonged to third‐grade classes, 30 students to fourth‐grade classes, and 55 students to fifth‐grade classes. Following a detailed explanation of the study design, written informed consent was obtained from all participants and their parents or legal guardians. The University Ethical Committee approved this investigation in accordance with the ethical principles of the Declaration of Helsinki (1964) and its subsequent amendments.

### 2.2 Experimental Design

All children participated in two experimental sessions, corresponding to two different types of PE lessons: a PE lesson without OC exercises versus a PE lesson with OC exercises. Saliva samples were collected immediately before (pre) and immediately after (post) both PE lessons. The sequential order of these lessons for each class was counterbalanced and randomized according to a predetermined plan, to control for potential effects on cortisol response due to the repetition of measurements. Experimental sessions took place at the same time of day (in the morning, before and after the scheduled weekly PE lesson) on the same school day each week, over 2 weeks. The school day was planned to follow the same schedule throughout the experimental intervention, in order to minimize the influence of diurnal physiological fluctuations in cortisol.

### 2.3 Measurements

#### 2.3.1 Salivary Cortisol Level

Salivary cortisol concentration was used to detect the acute stress response after exercise [28]. Children and their parents were advised to avoid any vigorous physical activity as much as possible, starting 24 h before the first saliva collection. Saliva samples were collected from all children using cotton swabs and saliva collection tubes (SALIVETTE, Sarstedt, Germany) immediately before and after the PE lessons. Contamination with food debris was avoided by rinsing the mouth with water and by delaying collection for 15 min after rinsing to prevent sample dilution. Salivary samples were then centrifuged at 2000 × g for 15 min (at 7°C) and stored at −40°C until analysis for cortisol concentration to evaluate the stress response to the PE interventions. Salivary cortisol concentration was measured by ELISA, using a commercial kit (DRG Instruments GmbH, Marburg, Germany).

#### 2.3.2 Exercise Intensity

During the PE sessions, exercise intensity was recorded using heart rate (HR) monitors (Polar S610i, Polar Electro Oy, Finland) to verify that the criteria for MVPA intensity were met (HR > 139 bpm) [29]. The HR monitors were set to alert when participants' HR fell outside the MVPA range, thereby ensuring that both types of lessons were delivered at comparable intensity levels and allowing for meaningful comparisons between the interventions.

### 2.4 Intervention

The two types of PE lessons were designed by the same specialist PE teacher, who delivered all lessons indoors. Both lessons were designed in accordance with the national curriculum guidelines for primary schools, which recognize school‐based PE as a fundamental and effective tool for developing physiological and fundamental movement skills and for promoting social interaction through the acquisition and practice of sport (DPR n.104 del 12.02.1985) [30]. The two PE lessons lasted a total of 50 min and were structured as follows: 15 min of warm‐up, comprising running at a slow pace followed by a progressive increase in speed, locomotor movements such as skipping and jumping with changes of direction, and sprint exercises at gradually increasing intensity; 30 min of continuous MVPA (with or without OC exercises, described below); and 5 min of cool‐down, comprising walking with deep breathing followed by stretching exercises.

The *PE lesson without OC exercises* consisted of a 15‐min continuous aerobic circuit‐training session with different coordinative exercises using basic gymnastics techniques: forward rolls on a mat, hurdle jumps at different distances and rhythms, cone slalom while performing different locomotor movements, and monopodalic and bipodalic hopping inside hoops placed at variable distances. This circuit training was followed by a 15‐min shuttle‐run game. The lesson focused on improving cardiovascular endurance through the performance of various basic motor patterns; all exercises were performed without rest, with variations in execution and intensity to maintain a target HR range.

The *PE lesson with OC exercises* consisted of the sport‐unspecific use of balls in the context of mini‐games. Using a basketball during locomotor movements, students were required to dribble and bounce the ball while walking, skipping, hopping, and running; to perform linear runs while dribbling; to catch and pass the ball to another student; and to repeat the same exercises with changes of direction. The same structure was then applied using a soccer ball, with object manipulation performed through foot control. This lesson aimed to enhance both motor control and perceptual‐motor adaptation skills, focusing on psychomotor competence and expertise in movement‐based problem solving, with functional use of the ball across tasks involving manipulative ball‐handling skills and decision‐making [31]. The ball was also used in an unconventional manner, to engage kinaesthetic differentiation, promote hand‐eye/foot‐eye coordination, and require precise timing, focused attention, and spatial adjustment skills [32].

### 2.5 Data Analysis

All results were expressed as mean ± standard deviation. A paired *t*‐test was performed to detect baseline differences in cortisol levels between the two PE interventions. Children's salivary cortisol levels were analyzed using a 3 × 2 × 2 × 2 mixed‐model ANOVA, with grade level and sex as between‐participants factors and time and activity type as within‐participants factors. The main effects of grade level (third vs. fourth vs. fifth), time (pre vs. post), activity type (without OC vs. with OC), sex (boys vs. girls), and their interactions were assessed, and significant effects were followed by Bonferroni post hoc analysis. We also calculated the change (Δ) in salivary cortisol concentration after each type of PE lesson compared to its initial value (post − pre), and analyzed this change using a repeated‐measures ANOVA with grade level as a between‐subjects factor and activity type as a within‐subject factor, followed by Bonferroni post hoc analysis where appropriate. Effect sizes were calculated using Cohen's convention for small, medium, and large effect sizes (partial *η* ^2^ = 0.01, 0.06, 0.14, respectively). Finally, mean and standard deviation of HR during the two PE lessons were compared using a paired‐samples *t*‐test. Significance was set at *p* ≤ 0.05, and analyses were performed using SPSS Version 27.0.

## 3 Results

No significant differences were observed in children's baseline cortisol levels between the two lesson types (without OC: 4.23 ± 3.26 nmol/L; with OC: 4.92 ± 3.60 nmol/L; *p* = 0.98). Table 1 reports the significant results relevant to the present study: the main effect of grade level (third vs. fourth vs. fifth), grade level × time and grade level × activity type × time interactions.

**TABLE 1:** Significant ANOVA results on cortisol level.

Factor	*F*	df	*p*	Partial *η* ^2^
Grade level	3.875	2	0.023	0.062
Time × grade level	7.627	2	0.001	0.114
Activity type × time × grade level	7.046	2	0.001	0.107

Post hoc analysis revealed significantly lower cortisol concentrations in fourth‐grade than fifth‐grade children (*p* = 0.02). The significant interaction between grade level and time revealed that only fifth‐grade children showed a significant cortisol decrease after the PE lessons (*p* = 0.01). The significant interaction among grade level, activity type, and time revealed a dissimilar trend by grade: third‐grade children showed a significant cortisol decrease after PE without OC (*p* = 0.045), with no significant change after PE with OC; fourth‐grade children showed a significant cortisol increase after PE without OC (*p* < 0.01), with no significant change after PE with OC; and fifth‐grade children showed a significant cortisol decrease after PE with OC (*p* = 0.03), with no significant change after PE without OC. Pre‐ and post‐intervention cortisol values for each grade level are shown in Table 2.

**TABLE 2:** Salivary cortisol concentrations (nmol/L) in children before and after both types of PE lessons in third, fourth, and fifth grades (means and standard deviations and their respective 95% confidence intervals for each group at each time point).

Grade level	PE without OC				PE with OC			
	Pre	95% CI	Post	95% CI	Pre	95% CI	Post	95% CI
Third	4.71 ± 3.17	[3.69; 5.74]	3.54 ± 2.12*	[2.86; 4.23]	3.10 ± 2.03	[2.44; 3.76]	3.22 ± 2.37	[2.45; 3.99]
Fourth	2.39 ± 0.88	[2.06; 2.72]	3.96 ± 2.22**	[3.13; 4.78]	3.21 ± 2.72	[2.19; 4.22]	2.56 ± 2.17	[1.75; 3.37]
Fifth^#^	4.23 ± 3.26	[3.35; 5.11]	3.46 ± 2.29	[2.84; 4.08]	4.92 ± 3.60	[3.95; 5.89]	3.85 ± 2.17*	[3.27; 4.44]

^#^ *p* < 0.05 fourth‐grade versus fifth‐grade.

* *p* < 0.05 post versus pre.

** *p* < 0.01 post versus pre.

Regarding sex differences in cortisol response, no significant main effect of sex, nor any significant interactions involving sex, were observed. Boys and girls showed comparable cortisol responses across both PE interventions.

The analysis of Δ salivary cortisol concentration revealed a significant activity type × grade level interaction (*F*_2,121_ = 6.884, *p* = 0.001, partial *η* ^2^ = 0.102). Specifically, only the Δ salivary cortisol concentration of fourth‐grade children showed significant differences between the two types of PE lesson (*p* = 0.003), revealing an increase in cortisol concentration after the PE lesson without OC and a decrease after the PE lesson with OC (Table 3).

**TABLE 3:** ∆ cortisol concentration (nmol/L) of children in third, fourth, and fifth grades in the two types of PE lessons (means and standard deviations and their respective 95% confidence intervals for each group).

Grade level	PE without OC		PE with OC	
		95% CI		95% CI
Third	−1.17 ± 3.53	[−2.32; −0.03]	0.12 ± 2.10	[−0.56; 0.80]
Fourth	1.57 ± 2.23	[0.73; 2.40]	−0.64 ± 3.52**	[−1.96; 0.67]
Fifth	−0.77 ± 3.91	[−1.83; 0.29]	−1.07 ± 3.62	[−2.04; −0.09]

** *p* < 0.01 with versus without OC skills.

Finally, HR data confirmed that both types of PE lesson were delivered at similar exercise intensity (146.56 ± 14.09 bpm for PE without OC; 147.25 ± 15.50 bpm for PE with OC; *p* > 0.05).

## 4 Discussion

This study aimed to investigate whether introducing OC exercises during PE lessons would influence the cortisol response of primary school children. The first finding was that primary school children showed significant variation in cortisol response after acute PE coordinative interventions, in agreement with previous studies reporting significant cortisol changes after cognitively and coordinatively demanding physical activity [16, 18], recognizing the role of this type of physical activity in eliciting a physiological response that may reflect stress‐related processes in this population [16, 18]. However, Budde et al. [19] did not find significant cortisol variation after high‐intensity aerobic exercise in primary school children; contrary to our study, their intervention did not include coordinative or OC tasks, which could explain the difference.

Salivary cortisol responses to physical activity in children should be interpreted with caution, as they reflect the interaction of multiple physiological and psychological processes beyond stress‐related mechanisms [33, 34], and cortisol regulation is influenced by multiple individual and contextual determinants [34, 35]. The reactivity of salivary stress and neurotrophic biomarkers in young cohorts has also been shown to be acute and sensitive to external rhythmic and acoustic environmental modulations, confirming that environmental stimuli can trigger immediate adaptive central and peripheral adjustments [36]. Within this multifactorial framework, the age‐related differences observed here may help clarify how children respond to different types of coordinative PE. The most notable finding of this study was that children of different grades showed distinct patterns of cortisol response depending on the type of PE intervention.

Specifically, the youngest children (third‐grade) showed a decrease in cortisol levels after the PE lesson without OC but not after the lesson with OC exercises: introducing a coordinative OC component did not produce the potential stress‐reducing effect typically associated with physical activity [37]. One possible explanation is that children aged 8–9 years are still developing foundational motor abilities, with mastery of fundamental movement skills generally not consolidated until approximately 10–11 years of age [14]. Younger children may also have a more limited capacity to differentiate their perceived competence across different types of OC skills [38]. Thus, for third‐grade students, the addition of OC exercises may have provided a more demanding physiological and cognitive stimulus, potentially preventing the cortisol decrease observed after PE without OC. This is consistent with Jäger et al. [18], who found that a cognitively engaging, playful MVPA intervention with OC tasks increased cortisol in children aged 6–8 years.

In contrast, fourth‐grade children showed a significant cortisol increase after the lesson without OC and no significant change after the lesson with OC. By this age range (9–10 years), many children have likely begun organized sport participation (e.g., mini‐volleyball, mini‐basketball, or soccer) [14] and may already have developed foundational ball‐control skills; the absence of OC content may therefore have reduced engagement and motivation, consistent with evidence that technical, task‐oriented goals (more prevalent in OC‐based lessons) are linked to increased motivation among young players [39]. This fits a broader psychophysiological view in which hormonal responses to physical activity are shaped not only by physical load but also by performance‐related stress and psychological states such as anxiety and peer comparison [35], so OC tasks may have increased cognitive and emotional demands particularly for children with lower perceived competence or less experience, influencing cortisol independently of exercise intensity. This interpretation aligns with broader evidence that hormonal reactivity is closely intertwined with emotional engagement and environmental stressors. Longitudinal and cross‐sectional studies of students during critical evaluation periods, such as examinations, have demonstrated clear correlations between academic stress, behavioral habits, and shifts in salivary cortisol profiles [40, 41, 42]. This is further supported by the Δ cortisol analysis, in which fourth‐grade children were the only group to show a significant inverse trend between lesson types.

Ishii and Osaka [43] similarly found that children who enjoyed PE were significantly less stressed than those who disliked sport, and that the latter did not benefit from the stress‐reducing effects of sport participation. This neuroendocrine baseline is also intertwined with children's internal cognitive strategies and psychological profile. Psychological screening tools evaluating emotional intelligence, emotion‐regulation skills, and metacognitive frameworks in developing children and youth have consistently revealed associations with salivary neuroplasticity markers such as BDNF and NDNF, and with corresponding cortisol fluctuations [44, 45, 46, 47, 48, 49]. A lower level of enjoyment during the OC‐free lesson may partly explain the cortisol increase observed in fourth‐grade children, although this remains speculative, as enjoyment was not assessed directly in the present study.

Fifth‐grade children showed the reverse pattern, with a significant cortisol decrease after the OC lesson but not after the lesson without OC. Using a longitudinal design, Wegner et al. [21] found that children who improved cardiovascular fitness over a 10‐week intervention showed higher cortisol, whereas those who improved motor coordination showed a decrease; the present fifth‐grade results (age 10–11 years) align with this pattern. Children with lower motor competence may perceive everyday motor challenges as more stressful, producing heightened stress responses, whereas more motor‐proficient children may experience coordinative tasks as more manageable and show attenuated cortisol responses [50, 51, 52, 53]. The cortisol decrease observed in fifth‐grade children, who are likely to have more developed OC skills than younger peers, may reflect this reduced stress response, although motor competence was not directly assessed. Exercise interventions that improve motor ability may also help preserve cognitive resources for coping with complex situations, further contributing to a reduced stress response [21]. Taken together, these findings suggest that developmental differences in motor competence across grade levels may partly underlie the age‐related cortisol patterns observed here, highlighting motor skill development as a moderating factor in children's physiological stress reactivity.

Overall, the youngest primary school children appeared to benefit more, in cortisol response terms, from PE without OC tasks, whereas older children benefited more from OC exercises. As children age, the introduction of ball‐control and OC mini‐games may be associated with more favorable physiological adaptation to physical activity. Miller et al. [11] found that OC competence was positively associated with game‐play competence and MVPA engagement in children aged 9–12, which may help explain why older children, who typically show greater proficiency in gross motor skills, coordination, and OC tasks [54], showed a cortisol pattern consistent with a stress‐reducing effect during the ball‐based lesson. Because proficient catching and kicking patterns tend to emerge around age 10 [12], the absence of a cortisol‐reducing effect in younger children during the OC lesson may partly reflect less developed coordinative ability. At the same time, OC exercises have documented benefits for younger children's motor development [55], so future research should explore how to introduce OC skill‐building without eliciting excessive stress responses tied to increased task demands.

A secondary finding was that fifth‐grade children showed generally higher cortisol concentrations than fourth‐grade children, which may reflect differences in basal cortisol or chronic physiological load. Fifth‐grade children also showed a more pronounced post‐lesson cortisol reduction. Although the relationship between chronic stress and age was not a study aim, Hanke et al. [17], studying children aged 10–13, reported that children with higher chronic stress benefit most from an acute physical activity session in terms of cortisol reduction, a pattern consistent with the present findings; though further research is needed to clarify the underlying mechanisms.

Several individual and contextual factors not assessed in this study, such as habitual physical activity, sport experience, motor competence, sleep quality, and emotional state, may have influenced the observed cortisol responses and children's perceived demands of the OC tasks [56, 57]. Cortisol responses in children are well documented to vary with interacting physiological and contextual influences [33, 57]; salivary cortisol should therefore be interpreted as an integrative marker of multiple concurrent processes rather than a direct indicator of stress alone.

It is also well established that salivary cortisol does not respond instantaneously, with peak responses typically occurring 15–20 min after a stressor's onset [58]. Because the present PE lessons lasted approximately 50 min, they likely represent a sustained stimulus rather than a brief acute stressor [18, 19, 59]. Cortisol activation may therefore begin during the early phases of the lesson, and the post‐lesson sample may still capture a meaningful portion of the hormonal response trajectory rather than a discrete peak. This immediate post‐lesson sampling approach is consistent with prior research using salivary cortisol as an indicator of exercise‐induced hormonal responses in school‐age children [18, 19, 60].

Overall, these findings contribute to the growing literature on children's physiological responses to PE, offering further insight into how coordinative demands interact with developmental stage to shape cortisol response patterns during PE lessons.

### 4.1 Implications for School Health Policy, Practice, and Equity

The findings of this study carry meaningful implications for PE curriculum design. Because the stress‐related effects of OC exercises differ by grade level, PE curricula should introduce OC tasks progressively, supporting both motor development and acute stress regulation across lessons. PE teachers should be aware that for younger children, particularly third‐graders, including OC exercises may attenuate the acute cortisol reduction otherwise associated with physical activity. This does not imply that OC tasks should be excluded from early primary PE, given their well‐documented role in motor development. Rather, teachers might embed such tasks within low‐evaluative, cooperative, exploratory (rather than performance‐oriented) formats to reduce the psychosocial stress associated with perceived motor incompetence among children who have not yet consolidated these skills. For older children in fifth grade, findings support a more deliberate use of ball‐based and OC mini‐games as both motor‐development tools and vehicles for acute stress regulation during PE. From an equity standpoint, because children's OC proficiency and prior sport exposure vary with socioeconomic and community resources, age‐differentiated PE planning that scaffolds OC exercises may help ensure physical activity remains health‐promoting for all students, regardless of existing motor competence. Teacher training programmes should incorporate this age‐ and competence‐differentiated approach to PE planning.

### 4.2 Limitations

Although the results of this study have added information about the relationship between OC skills and stress response in children, the study had some limitations. Measuring additional factors, such as children's perceived enjoyment, pleasure, feelings, and engagement during PE lessons, could have helped support our hypotheses, as these factors may affect cortisol responses. The study also did not assess children's physical activity level or their involvement in other sports, factors that could have influenced their stress response, as more active children may show no increase or only a slight increase in salivary cortisol after a physical stress stimulus [37].

## 5 Conclusions

The results of the present study suggest that primary school children exhibit different patterns of acute stress response following physical activity, depending on whether OC exercises are involved. Analyzing the overall findings across all age groups, the current data reveal a trend toward a more positive effect of physical activity interventions that engage OC skills as students get older. For younger children, who may not yet have fully developed complex OC skills, such tasks could potentially act as stressors, which might limit the stress‐reducing benefits of physical activity that are more readily observed in older children. Conversely, for older children, greater familiarity with OC demands may render such tasks more engaging during PE classes. Further longitudinal research is needed to help clarify the effects of different types of PE interventions on children's adaptability to stress. In this context, considering the progressive design of PE curricula alongside children's developmental status may significantly contribute to optimizing school health frameworks. Future studies should also consider the role of individual, psychological, and lifestyle‐related factors in shaping children's stress responses to physical activity to provide a more comprehensive psychophysiological understanding of these processes.

## Funding

Sapienza University of Rome, Grant number: RD1241910337879A.

## Ethics Statement

This study involved human participants and was approved by the University “Niccolò Cusano” Ethical Committee [Unicusano MO 6/22] in accordance with the ethical principles of the Declaration of Helsinki (1964) and its subsequent amendments. Written informed consent was obtained from all participants and their parents or legal guardians prior to data collection.

## Conflicts of Interest

The authors declare no conflicts of interest.

## Data Availability

The data that support the findings of this study are available on request from the corresponding author. The data are not publicly available due to privacy or ethical restrictions.
